# Erysipelas: New Methods of Treatment

**Published:** 1891-01-17

**Authors:** 


### ERYSIPELAS.—NEW METHODS OF TREATMENT.

Since it was demonstrated that a micrococcus was associated with the erysipelatous rash, and the probability of the dependence of erysipelas on the presence and vitality of this micro-organism was rendered almost a certainty by its inoculation producing the disease, many new and improved methods of local treatment have been advocated, nearly all being based on their germicidal action.

Now it is well known that suppuration and disorganisation of the eyeball follows destruction of the gasserian ganglion, but it has been shown that this will not occur provided the tissues be kept absolutely aseptic by the exclusion of the atmosphere and its contained putrefactive organisms, and the fact that the characteristic lesion in erysipelas is practically confined to the parts of the body exposed to the air would lead one to suppose that the specific organism only flourishes in the presence of the atmosphere, and clinical experience supports this hypothesis. We may thus explain the beneficial effects of the local application of collodion or flexible collodion and similar applications which act simply by protecting the part from the air. Nitrate of silver has been much used in times past for checking the progress of erysipelas, likewise glycerine of carbolic acid, applications which destroy the vitality of the micrococcus. We have recently had excellent results from the use of flexible collodion, containing biniodide of mercury (one part in two hundred and fifty, dissolved with iodide of potassium), an application which combines protection from the air with a powerful but non-irritating germicide. The ether of the collodion readily penetrates the epidermis carrying the germicide into the deeper layers of the skin. Ointments and greasy applications generally will hardly penetrate the epidermis, and therefore any germicidal agent they contain cannot act effectively. Our experience with this method is not extensive, but we believe that any case of erysipelas may be cut short in its earlier stages by this method, and that the exclusion from the part of putrefactive bacilli contained in the atmosphere will effectively prevent suppuration in the subcutaneous tissues. Mossbaum and Sir Dyce Duckworth use an ointment containing ichthyol. Koch recommends the application, by means of a camel hair brush, of a thin layer of an ointment composed of creolin one part, iodoform four parts, lanolin ten parts. The ointment is to be applied for some two or three inches of healthy skin around as well as over the erysipelatous patch, and then all is covered with a thin layer of gutta percha. In twenty-five cases thus treated a fall of temperature occurred, and the skin resumed its normal colour after two or three applications.

Nolte paints the entire surface of the affected part with a mucilage of gum arabic, containing three to five per cent. of phenic acid. Very excellent results were obtained by Rosenbach's method of washing the erysipelatous part and the surrounding skin with soap, then bathing the patch daily with a five per cent. solution of phenic acid, dissolved in absolute alcohol. Millihem has in three cases obtained an immediate and complete jugulation of erysipelas by painting with campho-phenique, two parts to one part olive oil. Hallopeau speaks favourably of the use of a solution of salicylate of sodium (one in twenty), thick cloths being wetted with the solution and applied, these being then covered with a layer of gutta percha or rubber cloth to prevent evaporation. Kingsbury recommends ergotine in equal parts of distilled water. He has found it a painless and almost certain cure. Hunter advocates the injection of carbolic acid in the healthy skin at a distance from the part affected, a method which unfortunately is very painful. Kraske recommends making incisions in the healthy skin around the patch before applying the antiseptic.

It is interesting that erysipelas, like some other specific diseases, has been made to serve as a remedial agent. Babchinski, while attending a very grave case of diphtheria, noticed a remarkable improvement in the patient's condition on the appearance of an erysipelatous rash on the face. This suggested to him the possibility of good results to be obtained by the inoculation of diphtheritic patients with blood from erysipelatous patients, and in several cases this treatment seemed to produce a cure. More recently he has found the inoculation of cases of diphtheria with cultures of the erysipelas microbe has proved equally satisfactory. We referred in a previous issue (Hospital, Aug. 9, 1890) to Dr. Kleeblatt's observations on the effects of erysipelas in cancer.

